# Visualized Characterization for Cerebral Response of Acupuncture Deqi: Paradox Underway

**DOI:** 10.1155/2013/894750

**Published:** 2013-07-02

**Authors:** Jie Yang, Ming-Xiao Yang, Fang Zeng, Xi Wu, Jiao Chen, Yan-Qin Liu, Yue Feng, Fan-Rong Liang

**Affiliations:** Chengdu University of Traditional Chinese Medicine, Chengdu, Sichuan 610075, China

## Abstract

Acupuncture as an oriental natural healing therapy with prolonged history has been extensively utilized in the management of great numbers of disorders. Deqi, a renowned acupuncture needling sensation, is profoundly regarded as the predictor and also the prerequisite of a preferable acupuncture treatment efficacy. Till now, there is still no consistency being reached towards the mechanism of acupuncture Deqi as a result of the discrepancy for publicly acknowledged evidence. Recent visualized research on Deqi using modern technologies has demonstrated possible central mechanism towards it. However, there is a conspicuous paradox underway in the research of cerebral response to acupuncture Deqi. This paper provided a view of up-to-date studies using visualized tools to characterize the brain response to acupuncture Deqi, such as functional magnetic resonance imaging (fMRI) and positron emission tomography/computed tomography (PET/CT). The paradox was extruded to highlight certain reasons from a TCM view. It is hypothesized that acupoints located at different dermal sites, state of participant, and needling manipulation can all contribute to the current paradox. Hence, further studies on acupuncture Deqi should pay more attention to the strategy of experiment design with generalized measurement, valid sham control methods, and more to subjects in diseased condition.

## 1. Introduction

In clinical practice, acupuncture is widely accepted as an oriental natural healing therapy with prolonged history and thus has been extensively utilized in the management of great deals of disorders both physically and functionally. The clinical effect of acupuncture has been greatly validated [1–4] and its potential mechanism is at the same time partially elucidated from different levels in recent decades, for instance, acupuncture analgesia [5–9]. Deqi, a renowned acupuncture needling sensation, is profoundly regarded as the predictor and also the prerequisite of a preferable acupuncture treatment efficacy [10]. This concept and belief has been imprinted in mind and highly esteemed to canonical principle in ancient times by both acupuncturists and patients ever since the very origin of traditional acupuncture in China.

As a subject adoring experience, the importance of Deqi in acupuncture is inheriting generation by generation in China. For supporting this point, there are always no less than of plentiful evidences in old times far away and at present. As the very initial classic of acupuncture in TCM, *Neijing* or *Huangdi*'s classics of internal medicine leaves us with a clue to its *Ling shu* (*Spiritual Pivot*) part, that is, “the essence of acupuncture is *Qizhi* (Qi arrival, another term commonly used as an alternative to Deqi in ancient times [11, 12]), it's always closely accompanied with satisfactory clinical efficacy”. Similar descriptions can be identified in the other part of *Neijing*, *Su Wen* (*Plain Questions*). In following ages, several symbolic works in acupuncture history also put emphasis for Deqi on the significance of acupuncture, like *Jin Zhen Fu* (*Ode to the Gold Needle*), and *Biao You Fu* (*Ode to clear Obscurity*) both embedded in *Zhen Jiu Da Cheng* (*Great Compendium of Acupuncture and Moxibustion*), *Medical Abecedarium* (*Yi Xue Ru Men*), *Zhong Guo Zhen Jiu Xue Shi (History of Chinese Acupuncture), Chinese Acupuncture Therapy *(*Zhong Guo Zhen Jiu Zhi Liao Xue*) written by Cheng Dan An*, Scientific Acupuncture Therapy *(*Ke Xue Zhen Jiu Zhi Liao Xue*) [11], *Zhong Guo Zhen Jiu Da Quan* (*Great compendium of Chinese acupuncture*) [13], and so forth. According to descriptions of the above books, the typical descriptors of Deqi sensation of a patient are soreness, numbness, distention, heaviness, and dull pain [14]. From a historical aspect, acupuncture Deqi refers not only to the sensation a patient may feel during an acupuncture treatment procedure but also to the acupuncturist's personal simultaneous finger feeling [15, 16]. To our knowledge, the most interesting and remarkable characteristic of acupuncture Deqi from the acupuncturist's view is a vivid metaphor as “fish bite the hook” in one's finger. As in the recent literature, little has been mentioned about the role of an acupuncturist's perception of Deqi, and the preponderance of acupuncture works underline various sensations that yield from acupuncture Deqi in a patient of both healthy and unhealthy conditions. Researchers in nowadays commence to question the credibility and importance of an acupuncturist's awareness of Deqi as to the clinical efficacy. However, we believe that it is nevertheless still disputable to assert whether acupuncture Deqi is monophasic or bipolar sensation.

At present, modern neuroimaging technologies are regarded as valid tools and have been widely applied to investigate the central mechanism of acupuncture effect. Deqi plays a central role in acquisition of acupuncture efficacy and thus recent CNS mechanism researches on acupuncture paid enormous attentions to it. In order to quantify the characteristics of acupuncture Deqi sensations, Kong and colleagues amended and modified the Subjective Acupuncture Sensation Scale (the SASS) to MGH Acupuncture Sensation Scale (MASS) for a precisely quantification of Deqi, and they also found close correlation between acupuncture analgesia for experiment-induced thermal pain and SASS rating of numbness and soreness instead of other Deqi descriptors such as stabbing, throbbing, tingling, and heaviness, in a pilot study comparing manual, electrical, and sham acupuncture efficacy [11, 17]. Hui et al. demonstrated in their works that the prominent components for characterizing acupuncture Deqi are aching, soreness, and dull pain; thus, according to the innervation of differed somatosensory conductance, it resulted in the conjecture that wide spectrum of myelinated and unmyelinated nerve fibers may play a noticeable role in the physiological response to acupuncture Deqi, particularly the slower conducting ones in the tendinomuscular [18]. But till now there is still no consistency being reached towards it as a result of the discrepancy for a publicly acknowledged standardized measurement. Furthermore, the main controversy is concentrated on the characterization of acupuncture Deqi with visualized approaches [19–26] along with behavioral manners [11, 18, 27–30], while most of these studies were performed on healthy subjects.

Based on this, in this paper we aim to provide a view to sum up-to-date studies using visualized tools to characterize the brain response to acupuncture Deqi, such as fMRI and PET/CT. The main body of the content was separately reviewed according to the differed points of view associated with the brain area state during the elicitation of acupuncture Deqi. Moreover, we would like to exhibit our suggestions for perspective studies in acupuncture Deqi. It is therefore expected by us that paradoxes and issues underlying current Deqi studies as we summarized in this paper could enhance the basis of understanding towards acupuncture Deqi, which consequently may supply better view angle to perform more influential Deqi studies focusing on cerebral response. In all, we hope that these studies may potentially contribute as solid evidence to support a more widespread usage of acupuncture both nationally and internationally.

## 2. Searching Strategy and Material Identification

As this paper focused on the cerebral reaction of acupuncture Deqi, hence we searched PubMed for articles in English language by both key words in title/abstract, “Deqi” AND “functional magnetic resonance imaging” or “fMRI,” “Deqi” AND “proton emission tomography-computed tomography” or “PET-CT,” “Deqi” AND “single photon emission computed tomography” or “SPECT,” “acupuncture sensation” AND “functional magnetic resonance imaging” or “fMRI,” “acupuncture sensation” AND “proton emission tomography-computed tomography” or “PET-CT,” and “acupuncture sensation” AND “single photon emission computed tomography” or “SPECT.” Totally, there was 23 articles retrieved. Within them, 7 articles were excluded for overlapping and 2 were eliminated for not a neuroimaging study (one article about expert opinion on Deqi and the other one only about behavioral index of Deqi sensations). As last, there was 14 articles included in the processing of review. 

## 3. Deactivation or Activation: Paradox Underway

fMRI and PET/CT are two common methods used in central mechanism study for acupuncture Deqi. On one hand, fMRI technology takes advantage of signal change of the blood-oxygenation level-dependent (BOLD) fMRI to reveal the hemodynamic response to measure the activity of brain tissues. That is to say, the signal intensity of BOLD fMRI reflexes the degree of blood O_2_ consumption by neural activity. BOLD signal increase generally demonstrates the redistribution of blood to activated brain zones with greater O_2_ demand which is regularly known as “blood stealing” or “physiological steal” [31]. On the opposite, negative BOLD signal often represents the neuronal deactivation or inhibition [32–34]. On the other hand, PET/CT and SPECT (single photon emission computed tomography) share similar paths to observe brain function. Through channels of rCBF, localized cerebral blood volume and metabolic rate of radionuclide in local brain region can be detected to indicate the condition of brain [35]. Mostly, FDG (fluorodeoxyglucose) is a frequent utilized tracer in PET study. Similarly, signal increase majorly indicates the high metabolism or blood flow in related brain and vice versa. Both fMRI and PET/CT are measures to evaluate the condition of brain functional activity. Indication of their signal change might share similarities with each other, and thus we to some extent regarded them as comparable.

In studies discovering the cerebral response by means of modern technologies, there is a stark paradox emerging regarding the state of certain brain regions participating in the response evoked by acupuncture Deqi. Besides peripherally characterizing acupuncture Deqi, Hui and colleagues conducted series-related studies and demonstrated that acupuncture Deqi in three acupoints evoked preponderant deactivations of several brain regions pertaining to LPNN (limbic-paralimbic-neocortical network), amygdala, and hippocampus [19, 20, 22, 36, 37]. In conspicuous contrast to this, other researchers discovered marked increase or activation of brain signals or brain activity responding to acupuncture Deqi. Hsieh et al. performed a PET study on acupuncture analgesia and found out that activation of the hypothalamus might characterize acupuncture Deqi in a commonly used analgesic acupoint, LI4, which was likewise utilized in Hui's study [38]. This finding is in consistency with a study revealed that Deqi sensation mainly resulted in marked activation of relevant brain areas covering Brodmann areas 6, 8, 19, 21, 28, 33, 35, 37, and 47, and parahippocampal gyrus, lentiform nucleus, claustrum, and red nucleus by carrying out a single-photon emission computed tomography (SPECT) on healthy subjects receiving acupuncture stimulation at TE5 [39]. In this part, we would demonstrate the above paradox underway in terms of brain response to acupuncture Deqi from the aspect of either deactivation or activation.

### 3.1. Deactivation Characterized Cerebral Response to Acupuncture Deqi

Recent research put emphasis on patient's subjective perception of acupuncture Deqi. In central mechanism exploration on acupuncture effect by method of visualized imaging technologies, specific brain areas responding to an activation or deactivation manner to acupuncture stimulation at fixed acupoints are firmly believed to serve as evidence to the effect of acupuncture. Studies on acupuncture analgesia demonstrated that acupuncture affected nociceptive matrix both specifically and nonspecifically, which entwined with the effect of expectations on the patient for pain relief [40]. Wu et al.'s fMRI study that tryed to shed light on the CNS mechanism of acupuncture analgesia demonstrated that analgesic acupoints (ST36, LI4) stimulation that elicited Deqi sensation might evoke deactivation of rostral part of the anterior cingulate cortex (ACC), amygdala formation, and hippocampal complex, though these areas were not the overall interaction of the brain with Deqi [41, 42]. That seemed quite not sufficient for the explanation of acupuncture Deqi mechanism, for the potential involvement of the subcortical gray structures and cerebellum. The structural basis of the CNS in response to acupuncture Deqi is commonly accepted as the limbic-paralimbic-neocortical network to date [19, 22, 36, 43].

Deactivated brain regions above involving the limbic systems are in agreement with Hui's finding on Deqi for the same acupoints. With stimuli at LI4, a frequently used analgesic acupoint, the prominent fMRI signal decrease was shown in the limbic system and several subcortical gray structures, including nucleus accumbens, amygdala, hippocampus, parahippocampus, hypothalamus, ventral tegmental area, anterior cingulate gyrus (BA 24), caudate, putamen, temporal pole, and insula. In subject with pain in replacement of acupuncture Deqi as well as superficial tactile control stimulation, the main signal change is not deactivated [19]. Other pieces of their series studies also implicated that other brain structures took part in the cerebral response to acupuncture Deqi. In another study stimulating ST36, multifaceted neural activities of the cerebrocerebellar and limbic system were modulated through differed level of acupuncture Deqi. Pure Deqi subjects demonstrated concerted attenuation of signal intensity in the limbic and paralimbic structures of cortical and subcortical regions in the telencephalon, diencephalon, brainstem, and cerebellum. But, in contrast to this, for subjects suffering from sharp pain together with acupuncture Deqi, the signal interspersed in the entire cerebrocerebellar and limbic systems was mostly increased. A hypothesis was proposed that the unique Deqi sensation leading to a deactivation dominated cerebral response might work in conflict with sharp pain, and their interaction with each other is in a dynamic equilibrium. Attention and anticipatory anxiety modulated brain response was also observed as deactivation for ventromedial prefrontal cortex abreasted with Deqi. In superficial tactile control, activation of the somatosensory cortices was induced just the same as that in pure Deqi subjects [22]. Identical finding was also demonstrated in other researchers' work [25, 44]. Hence, cerebral activities evoked by Deqi stimuli could be regarded as a multiple dimensional factor-guided response. Certain factors contributing to the peculiar cerebral response might include the unadorned substantial Deqi effect, attention, and anticipatory anxiety effect as well as tactile stimulation. To some extent, the magnitude of the deactivation of LPNN and the cerebellum is relatively minor, generally 1% [22], compared to 2%–4% of that in visual evocation [45]. Because the deactivation of LPNN caused by attention is less extensive than that of Deqi, then the demand of attention might be incomplete to explain the comprehensive signal attenuation of the entire brain. Their further study demonstrated that acupuncture may modulate the anticorrelated functional cerebral network to mediate its actions, and that the effect is relying on the psychophysical response [20, 24]. In another study carried out by Fang et al., similar salient response pattern was identified for Deqi produced by other points (*Taichong* (LV3), *Xingjian* (LV2), and *Neiting *(ST44)) other than Hui's points. It was deeply speculated that the involvement of these brain regions forming the Deqi responsive neural circuit and sole to the processing of pain in affective and cognitive angle as well as in modulation and integration of emotion, memory, autonomic, endocrine, immunological, and sensorimotor may act as potent evidence to support the idea that acupuncture Deqi is beneficial to the condition of being anxious, noxious emotions accompanied with pain [43]. Hence, it seems like stimulating different acupoint to achieve Deqi may to some extent produce similar brain response. Possible explanations were postulated in Section 4.

Napadow et al.'s study further compared manual acupuncture with electroacupuncture of varied frequencies and clarified that the limbic system was central to acupuncture effect of various acupuncture modalities, instead of merely manual acupuncture-induced Deqi [46]. EA with 2 Hz or 100 Hz with acupuncture Deqi achieved could, respectively, increase the fMRI signal of the limbic system as well as the manual acupuncture, though difference did exist in the brain areas such as both sides secondary somatosensory cortex, left inferior parietal lobule, and left dorsolateral prefrontal cortex. Relevantly, though without Deqi achieved, Han compared 2 Hz EA stimulation-induced brain area activation with that of 100 Hz for analgesic effect and demonstrated a frequency-dependent different, but overlapped, brain network. This may further supplement that different acupuncture modalities either with or without Deqi might yield different central response [47]. Duration of acupuncture Deqi may change the psychophysical feeling as well as the extensiveness and mode of central response. Li et al. showed that long duration (180 sec) manual acupuncture Deqi could enhance the deactivation mode and expand the extent of deactivated brain regions compared to short-term stimulation (30/60 sec) [48]. Thus, the central responsive domain and intensity of Deqi may be a time dependent and accumulative contributing factor to acupuncture therapeutic effects. An other study also demonstrated that acupuncture Deqi induced cerebral response with marked similarities of BOLD regardless of deep or superficial needling method [37]. It is therefore believed that these findings may further support the clinical practice for optimizing acupuncture modalities.

### 3.2. Activation Characterized Cerebral Response to Acupuncture Deqi

Brain areas involved in pain processing and perception are well aware typically such as SI (primary somatosensory cortex), SII (second somatosensory cortex), IC (insula cortex), thalamus, ACC, and PFC (prefrontal cortex) [49]. Amongst them, IC and SII are main interacting components of the sensory-discriminative dimension of pain, while ACC, thalamus, PFC, and posterior parietal cortices mainly compromise arousal and selective attention/orientating components of the attentional network for pain [50]. Regarding acupuncture Deqi, activation of several brain areas on the other hand in very few subjects was also presented in *Hui's* studies. For instance, subjects experiencing pain with nil Deqi sensation, or sharp pain with Deqi (mixed sensation) in acupuncture stimulation procedure exhibited activation dominated signal change of brain activity, and the brain areas involved were anterior cingulate gyrus (BA24), caudate, putamen, anterior thalamus, and posterior insula [19], which are frequently reported in correlation with pain perception. Thus, activation of these areas was interpreted as the mechanism of pain perception instead of Deqi. A trend similar to the above mentioned was noticed in the group of superficial tactile stimulation control. The overt activated brain region is the somatosensory cortex. From the aspect of cerebral network, acupuncture Deqi was demonstrated to regulate the default and somatosensory brain network to obtain an analgesic effect [51, 52] and also to modulate a larger spatiotemporal extent of spontaneous activities in the salient interoceptive-autonomic network, contributing to potential actions in the endogenous pain-modulation circuits and homeostatic control mechanisms [53]. Importantly, acupuncture Deqi's specific functional effect on CNS may leave over long-term influence through large-scale functional brain network [54].

There are also increasing numbers of studies disputing that acupuncture Deqi should induce evidently large activation of brain regions. Other studies manifested that classic acupuncture with Deqi could activate ACC, insula, cerebellum, superior frontal gyrus, medial and inferior frontal gyri, and amygdala which are in common with those activated by acute and chronic pain [55–57]. It indicates that the effect of acupuncture Deqi may regulate or even normalize the pain matrix of brain regions to a rectified status. Wu et al. shared the same view that acupuncture with Deqi achieved may activate structures as hypothalamus and nucleus accumbens of the descending antinociceptive pathway [41].

Despite these frequently administrated acupoints as ST36, LI4, and LV3 in Hui's studies, by achieving Deqi at other acupoints, a tendency of increasing activation over somatosensory-motor cortices, thalamus, anterior cingulate cortex, and insula areas was noticed with increasing EA parameters and Deqi response [42]. In a study on Deqi for Yuchi (LI11), the primary and secondary somatosensory area (SI, SII) and thalamus were observed to be activated by acupuncture Deqi signal, and postulation showed that the pathway of Deqi went via the stimulated-side thalamus into project to the stimulated-side S1. Several structures associated with emotion, sensation, memory perception, endocrine, and autonomic system activities were dragged in together to make Deqi considerably influential to emotional system, autonomic nerve system, and internal secretion system [58]. Besides, through activation of the sensorimotor cortices (BA 3, 4, 6, and 7) by Deqi, acupuncture also has been demonstrated to potentially amend the motor function of patients undergoing stroke by stimulating GB34 (traditionally used to recover motor function after several disabling diseases) in healthy patients [59]. Acupuncture has been demonstrated to be therapeutically beneficial to stroke patient not only for motor function recovery, but also for sensory rehabilitation. A study indicated acupuncture stimulation at LI4 accompanied with LI11 activated somatosensory cortex of stable somatosensory stroke patient. The somatosensory area was thought to be involved in mediating recovery from stroke via functional plasticity [60]. Zhang et al. attested in an fMRI study that stimulation of ST36/SP6 and GB34/BL57 in the same spinal segment yielded activation dominated response in some corresponding brain areas. Specifically, ST36/SP6 was able to activate orbital frontal cortex and deactivate hippocampus, while activating dorsal thalamus and inhibiting those of primary motor area and premotor cortex in GB34/BL57 [61]. Gareus et al. tried to determine whether or not a traditionally renowned vision-related acupoint could ignite the visual cortex; to their surprise, although the finding has shown inactivated BOLD response to visual cortex by Deqi in GB37, it did indicate the existence of clusters of activated voxels with significant activations in insula cortex, parietal operculum, parietotemporal cortex, inferior parietal lobule, superior colliculi, cuneus, middle occipital gyrus, and cingulate gyrus [62]. On the other hand, studies using other technologies of neuroimaging also connote similar activation in acupoints other than these commonly used in Hui's work. For instance, Chen et al.'s study of PET imaging has shown increased glycometabolism in Brodmann areas 6, 8, 19, 21, 28, 33, 35, 37, and 47, the parahippocampal gyrus, lentiform nucleus, claustrum, and red nucleus while the TE5 was punctured and Deqi was achieved [39]. Similarly, Yoo and colleagues found that acupuncture manipulation on PC6 inducing Deqi, in comparison to the sham acupuncture and tactile stimulation conditions, selectively activated left superior frontal gyrus, anterior cingulate gyrus, and dorsomedial nucleus of thalamus [63]. This finding is consistent with that of another study using PET technologies [64]. Hence, the varied dermal sites for needle stimulation could in a certain way lead to the paradox of brain response to Deqi underway.

As to different states of participantion, it also may result in the activated cerebral response to Deqi as reported in some studies. Activation of hypothalamus was also reckoned to be crucial to acupuncture Deqi in addicted patients and chronic pain as Carpal tunnel syndrome with stronger psychophysical feeling harvested in comparison with healthy subject [65, 66].

## 4. Discussion

Therefore, based on the summarization of neuroimaging investigation to acupuncture Deqi, it could be concluded that the paradox of visualized presentation of acupuncture Deqi-induced brain response is underlied in both fMRI and PET/CT studies. The main divergence is the pattern of brain response evoked by acupuncture Deqi. According to some studies, the preponderance of cerebral interaction with acupuncture Deqi is in a manner of deactivation in the LPNN brain system; on the contrary, other studies grasped the point that the prominent response of the brain to acupuncture Deqi is the activation in certain brain areas. The reasonable for this paradox is proposed as follows.

### 4.1. Explanation from Physiological Angle for Paradox in Deqi

Deqi as a key indicator of superior acupuncture efficacy is capable of evoking multifaceted cerebral response entwined with specific psychophysiological feeling. Several brain regions get involved in the acupuncture Deqi with certain responsive manner of activation or deactivation, such as the LPNN, cerebrocerebellar integration, and somatosensory cortex. Moreover, acupuncture stimulation with Deqi obtained may also participate in the normalization of the nociceptive brain network which is commonly regarded as the basis of pain perception regardless of acute or sharp pain. Previously, large majorities of studies have also demonstrated the mechanism of acupuncture analgesia from the viewpoint of peripheral nervous system [5–9, 67]. Four different kinds of endogenous opioids have been identified and attested to be mainly binding to the analgesic effect of acupuncture. Correlated to this finding, certain pathway from acupuncture analgesia effect has been formed. Mechanic signal of needling was sensed and transduced by sensorimotor sensor into psychophysical signal, which was then carried upwards by the excited A*β*/A*δ* and C afferent fiber [68–70] to the posterior spinal horn to further project into the brain via the spinal ventrolateral funiculus. Many brain nuclei composing a complex CNS network are involved in the processing of acupuncture analgesia. The Arc-PAG-NRM-spinal dorsal horn pathway is crucial to mediate acupuncture analgesia through the participation of the opioid peptides and their receptors (*μ*-, *δ*-, and *λ*-receptors) [68]. However, specific Deqi sensations have been dominantly characterized by studies as aching, soreness, and dull pain, which is prominently in close relationship with slow conducting A*δ* and C fibers. Thus deeper muscle layers with rich innervation of slow conducting fibers play an essential role in acupuncture Deqi though with all level nerve fibers participating [18].

### 4.2. Explanation from TCM Acupuncture Angle for Paradox in Deqi

In this paper, we demonstrated a considerable paradox existing underway in the neuroimaging research that tries to characterize acupuncture Deqi with visualized tools. The chief issue is encompassing the mode of response of brain regions in relevance to acupuncture Deqi. Some studies reported deactivation as the dominance and distinctive characteristic of Dei elicited cerebral response, while other debated activation to be that of Deqi. Inferring from the view of TCM, reasons are speculated and illustrated by us in this paper as followes.

(1) Acupoints located at different dermal sites possess varied abilities in function. From the aspect of TCM, meridians are internal and external linkage for viscera; different meridians are one-to-one corresponding to the internal organs. Acupoint on their circulating pathways can be utilized to modify abnormality of human body. Subsequently, their discrete function might cause different responsive brain regions, or similar reactive brain area with different modes. Furthermore, different acupuncture points are always believed to function subtly differently based on TCM theory. However, in real world practice, the peculiar treatment efficacy of certain meridian cannot be activated without relevant acupuncture point stimulation achieving Deqi. For instance, GB37 is traditionally applied to treat eye disease for the belief of its correlation with vision; neuroimaging studies also demonstrated specific activation of the visual area in brain by stimulating at it [71, 72]. Similar specificity has also been demonstrated in other points [73–75]. Thus, in previous studies on Deqi, one responsible factor for the paradox that could be posited is that different acupoints with their own unique efficacy could cause efficacy-related brain response which are certainly different in terms of pattern, mode, and intensity and as a result might contribute to the diversity of brain response to acupuncture Deqi. Therefore, it is understandable that acupoint with different body location and nerve innervation could induce varied cerebral response mode.

(2) State of participant is an influential factor to different brain response modes. As we mentioned in the above context, the bulk of the neuroimaging research on acupuncture Deqi was carried out on healthy subjects (HS). However, subjects in diseased condition may exhibit distinctive brain mapping in comparison with HS. For example, Zeng and colleagues demonstrated that in patients with functional dyspepsia, there is significant difference from that of HS. Certain brain areas that are key to determine the severity of symptom are the ACC, insula, thalamus, MCC, and cerebellum [76]. TCM doctors always emphasize the role of patient's condition in choosing optimal treatment methods. Basically, according to the description of *Neijing*, in the process of inducing acupuncture Deqi, the states of acupuncture receiver, in terms of excess or deficiency or both, are always vital to achieve a robust Deqi phenomenon. It is that people in an excessive state are mostly easier to get Deqi, while people in a deficient condition may hardly experience a Deqi effect. The differed cerebral response shall result from this probably. Based on their condition in difference, suitable modality of TCM as individualized treatment approach [77] will be prescribed for patient. On the other level, acupuncture classics also clarified that different health conditions may experience Deqi sensations varied from each other [11], and this may subsequently lead to the possible difference of cerebral response to Deqi. Therefore, it can be indicated that the incompatible responding manner of certain brain areas was potentially resulting from the varied health condition of patient.

(3) Needling manipulation may change the effect of clinical treatment and cerebral response. According to the textbook of Acupuncture and Moxibustion Administration Method, acupuncture needling manipulation can be generally categorized into the reinforcing skills and reducing skills, basically for relevant excess or deficiency body condition. These manipulations always include mechanic actions as lifting, thrusting, rotating, and stirring [14]. Mechanic stimuli of different nature apparently could lead to relatively special nerve activated and get specific sensation produced. This is in contraindication to a former opinion that mentioned that acupuncture Deqi with both superficial and deep needling produced similar activation and deactivation, rather than reinforced manner receiving deep needing as expected. A possible reason can be inferred from their report about the blending of Deqi with sharp pain (acute pain). In the deep needling group especially, there was a relatively high score of sharp pain together with Deqi sensation. As the sharp pain is deemed to act oppositely to Deqi and may reduce or weaken the magnitude and level of responsive pattern of brain regions, it is plausible that there are similarities for both deep and superficial needling. Thus, the presentation of cerebral response may in contradicted. Essentially, the sharp pain was confirmed as noxious stimuli for Deqi sensation, for it was posited that it was negative factor to neutralize the Deqi-induced therapeutic effect [22].

### 4.3. Suggestions for Future Deqi Studies

Based on the aforesaid reasons as issues for current research, we would like to express our opinion and offer our suggestion for forthcoming acupuncture Deqi research. As a result of the absence of experiments for subjects with disease, firstly we suggest conducting further studies on patients with control arm as HS and those with large population are needed to elucidate the acupuncture Deqi-induced cerebral effect [78]. Secondly, it is still necessary for researchers to form a workshop and hence to remodify the current nongeneralized quantitative measurements. The tool to quantify Deqi is very essential for characterizing the psychophysical traits of Deqi. Only with standardized acupuncture Deqi quantification we could set an equivalent baseline for the comparison with brain reaction mode and cerebral location of Deqi and nil Deqi. Thirdly, the design of a credible sham control is really important to qualify the nature of acupuncture Deqi. In order to acquire more reliable results, the methodological design is key to study of Deqi. Generalized manipulation methods and also placebo control means are also needed to attest the pure Deqi effect. Hence, forthcoming studies on acupuncture Deqi should pay more attention to the strategy of experiment design with generalized measurement, valid sham control methods, and more to subjects in diseased condition.
